# A Future of Words: Language and the Challenge of Abstract Concepts

**DOI:** 10.5334/joc.134

**Published:** 2020-10-23

**Authors:** Anna M. Borghi

**Affiliations:** 1 Sapienza University of Rome, Department of Dynamic and Clinical Psychology, IT; 2 Institute of Cognitive Sciences and Technologies, Italian National Research Council, IT

**Keywords:** Categorisation, Embodied cognition, Metacognition

## Abstract

The paper outlines one of the most important challenges that embodied and grounded theories need to face, i.e., that to explain how abstract concepts (abstractness) are acquired, represented, and used. I illustrate the view according to which abstract concepts are grounded not only in sensorimotor experiences, like concrete concepts, but also and to a greater extent in linguistic, social, and inner experiences. Specifically, I discuss the role played by metacognition, inner speech, social metacognition, and interoception. I also present evidence showing that the weight of linguistic, social, and inner experiences varies depending on the considered sub-kind of abstract concepts (e.g., mental states and spiritual concepts, numbers, emotions, social concepts). I argue that the challenge to explain abstract concepts representation implies the recognition of: a. the role of language, intended as inner and social tool, in shaping our mind; b. the importance of differences across languages; c. the existence of different kinds of abstract concepts; d. the necessity to adopt new paradigms, able to capture the use of abstract concepts in context and interactive situations. This challenge should be addressed with an integrated approach that bridges developmental, anthropological, and neuroscientific studies. This approach extends embodied and grounded views incorporating insights from distributional statistics views of meaning, from pragmatics and semiotics.

## Introduction

Embodied and grounded cognition (EGC) has spread widely in recent years. At its start, its impact was limited to a narrow group of pioneer researchers. Now it is a well-established theory (or collection of theories) characterized by different nuances, ranging from more enactivist approaches to less radical representational ones. Therefore, it is high time to solve some of the problems that have limited the impact of this view and to address more difficult challenges to render it more compelling (Ostarek & Huettig, 2019).

In this work, I will argue that in the future, it is important to ascribe to language, in its multifaceted dimensions, the role it deserves in shaping our mind. I will contend that attributing language an important role can help to address one of the most crucial challenges for embodied and grounded cognition, that to understand how abstract concepts are acquired, represented, and used (Borghi et al., 2017).

## Language and Embodied and Grounded Cognition

### Language as a pointer and as a shortcut

For many years, studies inspired by embodied cognition have investigated the grounding of concepts and language in the sensorimotor and then also in the emotional system (Barsalou, 1999; Gallese & Lakoff, 2005). The authors intended to reject the language-of-thought view (Fodor, 1975), and the idea that sensorimotor experience would be transduced in a semi-linguistic mental format. To counter this view, not only the role of language-of-thought but also the role of natural language in constraining cognition has been neglected. In this vein, theories of distributional semantics, according to which word meaning is derived by the co-occurrence of words, were considered as naturally opposed to embodied views, and as unable to grasp the conceptual meaning. The argument was simple, and, to me, still compelling: to grasp conceptual meaning, symbolic representations could not be grounded in “other meaningless symbols” but needed, instead, to be grounded in non-symbolic representations (symbol-grounding problem: Harnad, 1990). In the late nineties, some important theories of distributional semantics were proposed (HAL, Lund & Burgess, 1996; LSA, Landauer & Dumais, 1997). According to them, we would access word meaning referring to the linguistic contexts to which words were associated. For embodied cognition scholars, theories of distributional semantics were considered inadequate as theories of cognition and meaning. For example, Glenberg and Robertson (2000, p. 383) invited “to drop the assumption that meaning is based on abstract symbols arbitrarily related to their referents”, as hypothesized by theories of distributional semantics, and to focus instead on their grounding. LSA and HAL models were considered useful only as tools to test theories of meaning, for example, to decide among contrasting predictions based on simulation and based on word associations. In the first ten years of our century, the panorama was quite clear: embodied theories contended the camp with classical language-of-thought views on one side and with novel theories of distributional semantics on the other (e.g., Lund & Burgess, 1996; Landauer & Dumais, 1997). The main objective of EGC was to demonstrate that concepts and words evoked simulations and that they were not abstract, arbitrary, amodal: they were grounded in perception, action, and emotional systems rather than in linguistic information. Words were thus considered mainly as pointers that indexed their referents, that is as instruments, the main function of which was to refer to objects, entities, actions, situations (“pointers to simulations”, Barsalou et al., 2008, pp. 250). For example, according to the indexical theory (Glenberg & Robertson, 2000), language comprehension is constrained by the affordances of the linguistically mentioned objects. Things started to change in 2008–2010 when the role of language in shaping our conceptual representation started to assume more emphasis. The LASS theory (Language and Situated Simulation) (Barsalou et al., 2008) made an important step forward, arguing that both a linguistic system and a simulation one concur in representing knowledge. Importantly, the authors distinguished between linguistic forms and amodal symbols. While there would not be a system of amodal symbols that correspond to language, linguistic forms might constitute an important instrument to access meaning and index conceptual content. In the LASS view, word associations are particularly relevant in linguistic tasks, especially in the first processing phases, and work as pointers to the conceptual content. In the words of Barsalou et al. (2008, p. 251), “we assume that simulations represent deep conceptual information, unlike linguistic representations, which we view as more superficial.” According to the LASS theory (Barsalou et al., 2008), depending on the task, the linguistic system would exploit the brain modal systems or not; furthermore, the burst of linguistic associations would typically precede the slower, simulative process. In the same years, some models were proposed, which attempted to show that integrating sensorimotor and linguistic information could lead to a richer account of word meaning. Louwerse and Jeuniaux (2008) proposed that “language is both embodied and symbolic” arguing that embodied relations could be coded in the language, and ascribing a more crucial role to linguistic information. In a different but similar vein, Andrews et al. (2009) and Johns and Jones (2012) proposed that linguistic distributional and embodied models are not necessarily mutually exclusive. According to the first, “semantic representations are the product of what we call the statistical combination of experiential and distributional data types.” (Andrews et al., 2009, p. 464). Importantly, these two kinds of data are correlated. They can provide different information: the first source provides more information on sensorimotor features, the second on more abstract, encyclopedic knowledge. Johns and Jones (2012) built a model able to perceptually ground ungrounded words, in which the simulator of a word did not refer to real perceptual states but to perceptual states of associated words. The model was able to account for a variety of results in a variety of classical embodied cognition experiments. Andrews et al. (2009) showed that training a Bayesian model with both experiential data, derived from feature production tasks, and textual data, led to a richer representation of the knowledge of a set of words compared with both purely embodied and purely distributional models.

The linguistic shortcut view (e.g., Connell & Lynott, 2013; Connell et al., 2019) represents a mediation between two positions: according to the first language provides only a superficial way to access simulation (e.g., Barsalou et al., 2008), while according to the second language offers a rich medium to access meaning (e.g., Louwerse & Jeuniaux, 2008). The argument at the basis of the shortcut view is this: language can offer an economical and not costly way to arrive at the simulation. Hence, the role played by language is not marginal: language is a powerful means that reflects many aspects of the experience. Consistently, limited grounding is possible: in long term memory, concepts are grounded in sensorimotor and linguistic experiences, but, during their use, labels do not necessarily activate a simulation. In a similar spirit, in recent years, a variety of hybrid approaches as the previously described ones emerged. These approaches benefit from the insight of both distributional and embodied views, ascribing importance both to language and to simulation (e.g., Andrews et al., 2014). In a similar vein, Zwaan (2016) argues that sensorimotor and linguistic representations contribute and constrain each other in discourse comprehension. In this view, abstract concepts work as pointers that integrate previous information or information to come in a sensorimotor simulation.

To summarize: the fact that words make available knowledge beyond reference has been pointed out by many theories (e.g., MacDonald, Pearlmutter, & Seidenberg, 1994, among others). However, within embodied and grounded cognition, the focus of attention was on sensorimotor aspects, and less attention was devoted to language. The urgency to underline the role of grounding and simulation instead of language was initially owing to the need to contrast the dominant view, according to which concepts were amodal, and represented through propositional features. When embodied cognition started to spread, words were considered mainly as pointers to their referents. For years the focus of much research (more in embodied cognition than in grounded cognition) has been in finding how words activate their referents and the sensorimotor system. In recent years, words have been seen as providing a shortcut to meaning. In the first case, language was seen as having a shallow, superficial role, while, in the second, it was considered as a powerful way to access meaning, that on the fly could substitute simulations. Basically, instead of forming a simulation, for linguistic tasks, we can rely on the access to meaning granted to us by linguistic associations.

### A new way to think of language

Words are not only pointers to their referents and shortcuts to access meaning. I am not criticizing these views, neither the evidence that supports them, which I think is compelling and insightful. I am merely arguing that, in the future, embodied cognition views should focus more on the importance of language in shaping our mind, as highlighted by some new trends present in the literature. This change in focus does not imply undermining the role of sensorimotor and emotional systems. I will shortly address some of these views, arguing that words can work as physical tools, changing our perception of the environment and the body, as cognitive/inner tools, changing our way of thinking, and as social tools. I will address these three functions separately, but their distinction is blurred since language often plays these three functions concurrently.

#### Language as a physical tool

New studies are starting to show that language impacts perception of the objects (Gentilucci et al., 2003), influences object use, and can modify the perception of our bodily space, similarly to a lens or a physical tool. Language can, namely, provide us with a flexible instrument with which to formulate predictions, to be verified in light of the incoming sensory signals (Glenberg & Gallese, 2012; Lupyan & Clark, 2015). Language impacts visual and auditory perception: labels dynamically modulate visual processing (Lupyan & Spivey, 2010) and facilitate the detection of invisible objects (Lupyan & Ward, 2013; Ostarek & Huettig, 2017); furthermore, imagined speech affects auditory perception (Tian et al., 2018). The language also facilitates object use: In a recent study with immersive virtual reality (Foerster, Borghi & Goslin, 2020), participants interacted with six novel 3D tools, each associated to a novel way to functionally manipulate it (e.g., shaking the object and pressing a button to make it grow). Participants were taught a label to name three of them, while the other tools were left unnamed. EEG and behavioral timings were recorded when participants were required either to use the tool on a target-object, or simply to move it to a target-location. If labels simply contribute to the identification of tools, facilitation in both conditions should occur. We found instead that providing them with a label reduced the time to reach them, and speeded up and rendered more accurate the manipulations aimed at object use (but not at object transportation). Finally, similarly to physical tools (e.g., rake), words can extend our perceived near space (Scorolli et al., 2016). In recent years new studies have demonstrated that not only language, but also the different spoken (or signed) languages influence cognition. Neo-Whorfian approaches are gaining increasing consensus (e.g., Athanasopoulos et al., 2016). For example, studies have demonstrated that our perception of pitch, of odor, of color, of gender varies across the spoken languages (e.g., Dolscheid et al., 2013; Majid et al., 2018; Mazzuca et al., 2019, 2020; Winawer et al., 2007).

#### Language as inner tool

Language can extend our cognitive abilities (Clark, 1998; Dove, 2014, 2019): for example, it improves our procedural memory, it shapes our memory for time (Wang, & Gennari, 2019), it helps problem solving – more generally, it supports our thought. As Clark (1998) wrote, “the basic biological brain is fantastically empowered by some of its strangest and most recent creations: words in the air, symbols on the printed page.” (Clark, 1998, p. 15). One way in which language supports our thoughts is through inner speech. The notion was initially proposed by Vygotsky (1987/1934), according to whom an initially overt language becomes internalized and governs our thought. In recent years the interest for inner speech and its regulative, predictive, and mental wandering aspects has spread within philosophers, psychologists, and neuroscientists (Langland-Hassan & Vicente, 2019; Alderson-Day & Fernyhough, 2015). Inner speech provides an embodied way to think about our thinking process; it is a predictive process that involves a form of motor simulation and that engages prefrontal and motor cortices involved during overt speech (Tian & Poppel, 2012).

#### Language as a social tool

Many actions in the social environment occur through language. The idea that language is a kind of action that modifies the social environment has a long tradition in pragmatics (Searle, 1985). Still, its consequences could be further explored in an embodied perspective. The conversation is the outlet where the social nature of language has one of its maximal expressions. The studies that interpret dialogue and conversation as a kind of joint action (Galantucci & Sebanz, 2009; Pickering & Garrod, 2013) highlight the fact that language is a social tool. Research on experimental semiotics and experimental pragmatics, and new neuroscientific work on brain-to-brain coupling that allow researchers to investigate conversational dynamics (Hasson et al., 2012) represent promising areas to study language and its social dimension.

### Summary

Interesting new trends are emerging that highlight the importance of language for our cognitive processes. Words work as pointers, and they can provide shortcuts to meaning, as embodied theories claim – but this is not the whole story. Linguistic associations allow us to capture part of embodied meaning, as hybrid embodied- distributional semantics theories claim. Again, this is not the entire story. I think that embodied and grounded cognition approaches will succeed if they will be able to recognize the role of language, both external and inner, as a predictive device that shapes our mind, and to capture the flexibility that characterizes language in context, promoting the use of paradigms that involve two or more participants and that aim to capture the dynamics of real conversations and of linguistic interactions.

## Abstract Concepts and Embodied and Grounded Cognition

In this section, the structure of which mirrors that of the first part, I illustrate why I think that the role of language is of paramount importance to address the challenge of abstract concepts. Following the schema of the first part, I outline how the idea that language can work as an inner and a social tool influences abstract concepts representation. I then highlight the necessity to consider not only language, but the differences across languages and to design and use new, more interactive methods that take into account the social dimension that accompanies language. Finally, I will argue that, the more abstract concepts are, the more crucial the role of language is. It is therefore primary to investigate the differences between kinds of abstract concepts, as it has already been done for concrete concepts (Figure 1).

**Figure 1:**
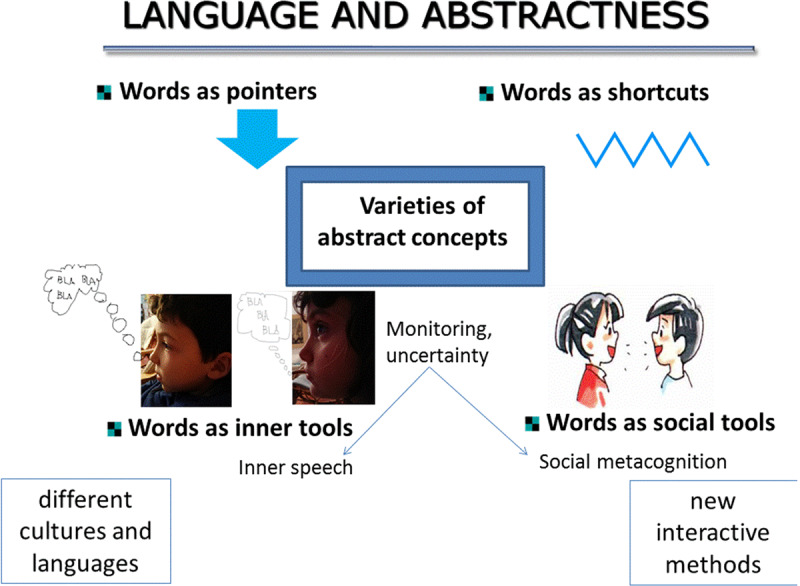
Language and abstractness: words can work as pointers, shortcuts, inner and social tools. New research should consider in-depth linguistic differences and adopt new interactive methods to investigate conceptual use.

Before starting, it is essential to define abstract concept, to explain why they can represent a challenge for embodied cognition and to clarify which view of grounded cognition we adopt.

### The challenge of abstract concepts

One of the most sophisticated human abilities consists of proficiently using abstract concepts, such as “freedom”. Consider some commonly used English corpora: 59% of the words of the SUBTLEX movie subtitle corpus (Brysbaert & New, 2009) are rated above the mean of abstractness (e.g., “immodest”); if you consider the norms of Juhasz and Yap (2013) on sensory and perceptual experience in words, the probability of picking up a word rated above the median level of abstractness is 74% (Lupyan & Winter, 2018). Hence abstract words represent a large part of our word use. How are we able to acquire and use them, even if they have been called “hard words” (Gleitman, 2005)?

Explaining the ability to use words that do not map to a concrete, single referent represents a real challenge, especially for embodied cognition (Borghi et al., 2017). Similar to concrete concepts, also abstract ones are grounded in sensorimotor experiences and activate a variety of situations. However, demonstrating their grounding compellingly is not easy. Notably, most evidence on embodied cognition concerns manipulable objects, action verbs, simple sentences that include action verbs.

A strong embodied theory would not assume any difference between concrete and abstract concepts. According to such a view, both concrete and abstract concepts would be grounded in bodily states, that is in the sensorimotor systems, and eventually in the emotional and interoceptive dimensions. We assume instead that to fully account for abstract concepts an extension of embodied cognition is necessary. Indeed, concepts might be grounded in ways that go beyond the sensorimotor system and bodily states; more specifically, different kinds of grounding might have a different weight for concrete and abstract concepts. Sensorial and bodily experiences might be more crucial for concrete concepts, and social and linguistic experiences for abstract ones. Here I adopt the definition of grounded cognition proposed by Barsalou (2008) “Grounded cognition reflects the assumption that cognition is typically grounded in multiple ways, including simulations, situated action and, on occasion, bodily states”.

As I will detail below, abstract concepts can be grounded in a variety of experiences. Some of these experiences involve the body primarily – for example, I will show that interoceptive experience is crucial, especially for some kinds of abstract concepts, the emotional ones. Some of these experiences might also involve the body, but not only the body. It is the case, for example, of the linguistic experience: it has an embodied counterpart, the activation of the mouth motor system, but it cannot be reduced to it. I contend that, to fully account for abstractness, not only bodily experiences, be they sensorimotor or interoceptive, play a role, but that social and linguistic experiences have crucial importance, and that their role differs depending on the considered kind of abstract concepts.

The most interesting theoretical advance in recent years is offered by multiple representation theories (e.g., Dove, 2009), that start from the consideration that multiple systems concur in explaining conceptual representation; for abstract concepts, not only sensorimotor experience is considered crucial, but also emotional, social and linguistic one. Within multiple representation views, the Affective Embodiment Account (AEA) highlights the relevance of affective experience. It proposes that abstract words evoke, more than concrete ones, emotional experiences (Kousta et al., 2011; Vigliocco et al., 2014). Furthermore, emotional words can provide a bootstrapping mechanism to learn abstract words, since they are the first words without a concrete referent acquired by children (Lund et al., 2019; Ponari et al., 2017). Here I will focus on multiple representation views that, although being embodied cognition ones, take into account an important lesson from theories of distributional semantics, the relevance of language (Dove et al., 2020). As we have seen, some theories have shown the advantages of combining experiential and distributional data in accounting for conceptual knowledge. Furthermore, they have emphasized the fact that abstract, encyclopedic knowledge might be particularly relevant for abstract concepts (e.g., Andrews et al., 2009; Johns & Jones, 2012). According to the Words As social Tools (WAT) view (Borghi & Binkofski, 2014; Borghi et al., 2019), language, sociality, and inner grounding actively contribute to determine the meaning of abstract concepts. WAT puts a strong emphasis on the fact that, because abstract concepts are less constrained by the environment than concrete ones, their acquisition is strongly mediated by linguistic and social inputs. Hence, compared to the previously discussed theories that integrate experiential and distributional data, WAT puts a strong emphasis not only on linguistically encoded semantic associations but also on the linguistic experience as a whole, considering its pragmatic aspects and the social dynamics that characterize it (for details on the theory, see Borghi et al., 2017, 2018a, b). For example, while processing abstract concepts, we might need and rely more on the help of other people (Borghi et al., 2018; Fini et al., under review). WAT also contends that such a form of acquisition influences conceptual representation, rendering linguistic and social neural networks more paramount for the neural representation of abstract than of concrete concepts. Directly linked to the WAT proposal is the LENS (Language as an Embodied Neuroenhancement and Scaffold) theory, which sees “language as an external symbol technology that becomes an integrated part of the neurologically realized conceptual system” (Dove. 2019, pp. 3).

To what extent does WAT depart from a grounded theory such as the Theory of Perceptual Symbols (PST) (Barsalou, 1999)? Barsalou’s 1999 article was and is a crucial and rich source of inspiration for me and many others, and in that seminal paper the author developed or at least mentioned many of the issues pointed out in elaborating the WAT proposal. Barsalou (1999) identifies three mechanisms for representing abstract concepts. First, abstract concepts would be framed in the context of extended event sequences (framing). Then, selective attention would select their content in the context of the extended event (selectivity). Finally, to represent them, perceptual symbols for introspective states would be central (introspective symbols). Barsalou (1999) shows how these mechanisms can account for the core sense of concepts like truth, falsity, anger, and negation.

The WAT proposal is a grounded one; hence it assumes the principles outlined by the Theory of Perceptual Symbols. Building on it, WAT starts from the assumption that selecting a portion of an event is more complicated than to focus on an object or a part of it. It thus underlines the role the help of other people has to select the right portion of events to which concepts refer. Labels and explanations of other people are fundamental to form categories, like “justice” that keep together a variety of heterogeneous exemplars. WAT does not assume a dichotomy between concrete and abstract concepts: for example, it hypothesizes that the role of language is more prominent for more abstract concepts, such as the religious and philosophical concepts (e.g., “belief”, “philosophy”), and less for more “embodied” abstract concepts, like emotional ones (Villani et al., 2019a, b).

PST also recognizes the role of language, suggesting, for example, that “perceptual symbol systems are continuous across development, with the addition of linguistic control added to achieve social coordination and cultural transmission.” (Barsalou, 1999, p. 607). However, its focus is more on sensorimotor experience: « If infants have the schematic symbol formation processing described earlier, and if they can apply it to their introspective experience, they should acquire an implicit understanding of truth, falsity and negation long before they acquire language. » (Barsalou, 1999, p. 602). In 2008, Barsalou et al. (2008) wrote that, since many had focused on the importance of language, it was essential to emphasize sensorimotor grounding. I think that now many empirical demonstrations have shown the importance of perceptual symbols and sensorimotor grounding, and the time has come to put a new emphasis on the role of language, intending language as a form of experience, in a grounded perspective.

The role WAT ascribes to inner monitoring and metacognition is also indebted to the notion of introspection in the processing of abstract concepts outlined by Barsalou (see Borghi et al., 2017, for discussion on this). According to WAT, when processing abstract concepts, we would detect through inner monitoring that our knowledge is not adequate; this uncertainty on their meaning would lead us to implicitly or explicitly ask the contribution of others (social metacognition).

As to cross-linguistic differences, also PST takes them into account, arguing, for example, that there might be similarities, across cultures, in the way we conceive introspection. In line with this view, we agree that what we call monitoring, and metacognitive aspects might be important mechanisms that hold for abstract concepts in general, across cultures (see Borghi, Tummolini & Fini, in press). However, the WAT view deals with cross-linguistic differences, also arguing that, because language is so crucial for abstract concepts, these last are more likely to be influenced by linguistic differences and have a higher probability to vary across languages compared to concrete concepts.

Hence, starting from a grounded view, WAT 1) underlines the role of conceptual acquisition, and hypothesizes that, because the social and linguistic input is crucial for abstract concepts acquisition, it might also influence brain representation; 2) hypothesizes a different distribution and role, in concrete and abstract concepts, of sensorimotor vs. linguistic, social and inner (interoceptive, metacognitive) experiences; 3) stresses the function of sociality, emphasizing the importance of the contribution of other people, primarily to support the acquisition but also the use of abstract concepts (social metacognition); 4) highlights the fact that, because of the role of overt and inner language for abstract concepts, their processing might involve the mouth motor system; 5) hypothesizes that, because the linguistic input is crucial particularly for abstract concepts, the meaning of abstract words is more affected by cross-linguistic differences.

It could be objected that a more parsimonious way to formulate WAT would consist in arguing that concrete and abstract concepts differ because they are anchored to different kinds of situations. While this general claim is undoubtedly valid, WAT specifies this claim further. Compared to a theory that proposes that abstract and concrete concepts are linked to different situations, WAT:

1. qualifies the kinds of situations, arguing that linguistic and social inputs are typically more crucial for abstract than for concrete concepts, for which the sensorimotor input is more relevant;
2. refers not only to situations related to the content of the concepts but also to their acquisition and use.

To clarify: the concept « logic » might evoke situations in which a person is speaking to others and uses a consequential and logical argumentation. Hence, this concept would evoke social and linguistic situations owing to its content. We think, however, that compared to concrete concepts abstract ones need more linguistic and social inputs during their acquisition and use: for example, because the members of the abstract concept « logic » are very sparse and heterogeneous, other people (or also written sources) are more needed to help the learner to understand the word meaning, and in supporting him/her to identify the correct one during online word use. For example, during on the fly conceptual use, compared to concrete concepts, abstract ones might require more social skills to identify authoritative sources that can support us and more social coordination with others. Hence WAT underlines the role of language and sociality not only in situations linked to the conceptual content, that become part of the long-term memory storage anchored to it (Connell & Lynott, 2016), but also in situations related to the conceptual acquisition and to on the fly word use.

Before summarizing the content of this section, an observation. It could even be argued that the proposal that language plays a significant role in abstract concepts does not avoid Harnad’s (1990) symbol grounding problem. I do not think that this problem is present. We do not deny that words point to their referent, and neither that they can provide a shortcut to meaning. Hence, the symbol grounding problem is avoided. We argue, however, that words are not only pointers and shortcuts, and that word use play a role. We thus need to consider their many possible associations to and combinations with other words, as theories of distributional semantics do. We also need to take into account word use during interactions with oneself (inner speech) and other people, and the social context in which they are acquired and processed. In sum: each word carries with itself broader experiences, which are not limited to the fact that the word points to its referent(s) – and yet, it points to its referent(s). One could ask how could abstract words indicate their referents, since their referents have no physical manifestations. Notice that I would not endorse grounded cognition theories if I believed that the referents of abstract concepts have no physical manifestation (Cuccio & Gallese, 2018). I am saying instead that they have a variety of referents, that these referents generally do not correspond to bounded objects, and are typically more variable across participants and situations than those of concrete objects. For example, “freedom” might refer to the situations of observing rough sea, running on a beach, and flying like a bird.

### The role of language in abstractness ratings

Abstract concepts were traditionally defined in a negative way, highlighting the properties they do not possess when compared with concrete concepts. They were described as more difficult than concrete concepts, more detached from perceptual modalities (Barsalou, 2003) and as referring to a collection of heterogeneous situations and events and not to a single, clearly identifiable object/entity. Now it is accepted that the distinction between abstract and concrete concepts is not dichotomous (Wiemer-Hastings et al., 2001), and it is a simplification since all concepts, even the most abstract (concrete) ones, can have concrete (abstract) components, when activated in context (Barsalou et al., 2018). In recent work, we have proposed that concrete concepts are more grounded in the sensorimotor and exteroceptive experience, while for abstract concepts language, sociality and inner experiences play a major role (Borghi et al., 2017, 2019). Barsalou et al. (2018) proposed either to drop the term abstract and concrete or to redefine them, taking into consideration that concepts are useful for situated actions. Concrete-STF (Situated Conceptualization Framework) originate in external elements of situations, such as settings, objects, and actions. Abstract-STF include instead either concepts originated in internal elements of situations, such as emotions, mental states, and goals, and concepts aimed to situational integration among situational elements.

But how do people represent abstract concepts? Is linguistic experience an integral part of their representation? In a recent study (Villani et al., 2019), we asked participants to rate 425 abstract words on a variety of dimensions. These dimensions included the most commonly used in the literature, such as abstractness, concreteness, imageability, context availability, but also many novel dimensions related to perceptual strength, inner grounding, and language. They were emotionality, interoception, Body Object Interaction, metacognition, social metacognition (need to rely on other’s knowledge), mouth and hand activation, Perceptual modality strength (five senses), Social Valence, Age of Acquisition, Modality of Acquisition. We ran a parallel analysis based on a Principal Component Analysis (PCA) and identified a three-component solution. We named the components “Concreteness/Abstractness”, “Inner Grounding and Social” (it included metacognition, interoception, emotionality, social dimension and mouth effector), “Sensorimotor” (it included sensory modalities and the hand activation). Our analysis suggested that a multidimensional feature space exists, in which many dimensions together determine whether a concept is more concrete or more abstract (see for similar views Crutch et al., 2013; Troche, Crutch, & Reilly, 2014; Harpaintner et al., 2018). Crucially, the analysis of the first component (Abstractness/Concreteness) allowed us to understand how abstractness was conceived: it was characterized mainly by linguistic components, that is by the late Age of Acquisition of the words, linguistically mediated acquisition (MoA), and high reliance on knowledge of others (social metacognition), and it contrasted with concreteness because of the low scores in Body Object Interaction, context availability, and imageability. Linguistic MoA, early AoA, the strong need of help of others to capture meaning are the most crucial aspects that characterized abstract concepts, followed by dimensions related to inner grounding (emotionality, interoception, metacognition, social dimension, mouth activation), which however formed a separate component. Hence, language characterizes how we represent abstract concepts.

### Language as inner and social tool for abstract concepts

When we learn new concepts, we benefit of linguistic information more with abstract than with concrete concepts (Granito et al., 2015): the members of the first are more varied and heterogeneous, and we need more the input of others to explain us how different exemplars can be tied; furthermore, words can help us to put together perceptually sparse experiences. This acquisition modality, along with the more marked role of inner experience with abstract concepts, has an influence also on their brain representation. Here I propose that language works as an inner and social tool during abstract concepts use (this does not exclude that it can also be used as a pointer and a shortcut to meaning). I will shortly outline different, but not conflicting mechanisms, that in the WAT view are particularly crucial for abstract concepts processing: they are metacognition, inner speech, and social metacognition. We hypothesize that an embodied outcome of these mechanisms is the engagement of the mouth motor system. In different studies with children and adults, we have found evidence that, with abstract concepts, the mouth motor system is activated (for a review see Borghi et al., 2019). We found either a facilitation of mouth responses during abstract words processing (e.g., Borghi & Zarcone, 2016; Mazzuca et al., 2018) or interference when a device impeded the active mouth use; for example, we found that prolonged use of the pacifier in children might set a footprint on abstract concepts processing (Barca et al. 2017, 2020). Lastly, I will briefly refer to another dimension, not directly related to language but important for inner grounding of abstract concepts, that of interoception.

#### Monitoring

Because abstract concepts are quite complex and characterized by heterogeneity of their members, we propose that the monitoring process of metacognition is more associated with their use than with that of concrete ones. We think that this monitoring process, accompanied by a feeling of uncertainty and scarce confidence in the word meaning, can lead to different outcomes. The first is a longer internal search process, likely mediated by inner speech (Borghi, Fini, & Tummolini, in press). Signals of it are the perceived greater difficulty of abstract words, and the longer processing time they require compared to concrete ones (concreteness effect).

#### Inner speech

This inner search can occur in different ways, for example, listing possible meanings, repeating the word, or re-explaining the meaning to ourselves. In an embodied perspective, we propose that this process occurs (mainly) through inner speech, and that involves both phono-articulatory and semantic components. As a consequence, the mouth motor system would be engaged. Notice that we do not think that the mouth motor system linked to the (inner) language is activated because it bears any relation to the content of abstract words. Instead, we think it is activated because (inner) language can help us in monitoring our knowledge, in searching for meaning, and in internally elaborating it, and for the reasons above these operations are particularly crucial for abstract concepts. In line with other theorists (Vygotski, 1987/1934, Wilkinson & Fernyhough, 2018), we conceive inner speech as a form of real speech, that is a phenomenon that leads to the production of speech acts. Inner speech is both sensory and motor; it does not involve only auditory imagery, but also motor imagery and, importantly, the sense of agency (Perrone-Bertolotti et al., 2014; Wilkinson & Fernyhough, 2018). At a neural level, inner speech processing is compatible with the selective activation of the left inferior frontal gyrus (LIFG) during abstract concepts processing (Wang et al., 2010; Binder et al., 2009). LIFG is typically engaged for phono-articulatory processing and working memory: our higher uncertainty with abstract words could generate the need to maintain them longer in working memory. A recent article confirms that during abstract thought, areas linked to inner speech, intended as a form of covert linguistic production, are activated (Berkovich-Ohana et al., 2020). In keeping with the hypothesis that abstract concepts processing involves inner speech, in two recent experiments, we found that articulatory suppression disrupted the processing of abstract concepts. Response times in categorizing words as concrete/abstract with abstract words were slower when participants performed a concurrent articulatory suppression task (continuously pronouncing a syllable) than a concurrent manipulation task (rhythmically manipulating a softball); this did not happen with concrete words (Zannino, Fini, Benassi, Carlesimo, & Borghi, under review).

#### Social metacognition

The second outcome of the feeling of scarce confidence is external and leads to a process that we called social metacognition (Borghi et al., 2018). This process is similar to what Shea et al. (2014) call System 2 metacognition (see also Shea, 2018), but we hypothesize that it has an embodied counterpart, the mouth motor system activation. The idea is that, because we are metacognitively aware of the complexity of our concepts and are scarcely confident in accessing their meaning, we would revert to other people, possibly choosing authoritative informants. Whether this process is implicit or explicit has to be determined. It certainly has an explicit outcome, the tendency to rely more on others. We believe that the evidence we found on mouth motor system activation is compatible with such a mechanism – the tendency to ask others and to prepare ourselves to do it would be stronger with abstract than with concrete concepts. When using abstract concepts, we might thus be more aware of the fact that knowledge can be distributed across different heads, and the ability to discriminate among reliable and not reliable informants might be more important. Among the reasons for the later acquisition of abstract words, there might be the limited ability of young children in selecting the right informant and in discriminating between real and pretend competence (Kominsky et al., 2016). Recent evidence (Fini, Era, Darold, Candidi, Borghi, 2020) revealed that, when participants were presented with pictures that displayed complex situations, they made more errors when they had to guess abstract rather than concrete concepts, and had to ask more frequently suggestions to the experimenter. Later they were required to perform a joint action task with an avatar, grasping a bottle in an imitative or complementary fashion. A further conceptual guessing session would follow the joint action. Participants were told that the avatar embodied the movements of the confederate who would give them suggestions on abstract concepts, or by the one who would give them suggestions on concrete ones. Participants were more synchronous in their movements with the avatar led by the experimenter who gave them advice on abstract concepts, consistently with our hypothesis: because with abstract concepts, we are less confident, we need more the help of others; hence we tend to be more collaborative with them when performing a joint action task.

#### Interoception

Interoception is the awareness of the signals coming from the body. It is one of the frontiers embodied cognition will have to face. Here I will not focus on interoception per se but on its relationship with abstract concepts. Connell et al. (2018) have shown that interoception is particularly crucial for abstract concepts, especially negative emotions. In recent work (Villani, Lugli, Liuzza, Nicoletti, Borghi, 2021), we asked participants to rate the difficulty of concrete and abstract words and to perform a concurrent task. This task consisted in chewing a gum, pronouncing a syllable, determining how good they were in counting their heart beating, or squeezing a softball. Difficulty can be a signal of confidence in accessing the word meaning, and we predicted that perceived difficulty would increase with an interferent condition that tapped on a dimension relevant for the representation of the concepts to evaluate. Consistently with our predictions, we found that the interoceptive condition influenced more abstract concepts, and particularly emotional abstract concepts, leading to an increase of the difficulty ratings, hence of the perceived difficulty of words.

To summarize: In this part, I have highlighted some possible mechanisms that underlie abstract concepts processing. We have posited that the mouth motor system activation found in a variety of studies might be due to a longer monitoring process, which leads to a long search for a possible meaning. We have proposed that this search for meaning might occur through inner speech, thus involving the phono-articulatory system. This mechanism implies an active use of working memory. As explained by Binder et al. (2005), the activation of the left IFG can be accounted for by the long search for meaning in working memory. A possible different mechanism that could coexist with the first or not leads to an outer search: we would prepare ourselves to ask information on the word meaning to others (social metacognition). The distinction we posit between these two different ways to employ inner speech could be consistent with the distinction between a monologic and a dialogic form of inner speech, with the second engaging a broader and more bilateral network of neural areas that go beyond the left frontotemporal regions (Alderson-Day et al., 2016).

Other mechanisms can explain the mouth motor system activation, and they can even coexist with the ones we propose. For example, because, with abstract concepts, it is quite difficult to associate the referent with a mental image, we could strategically use the phonological loop of working memory as a memory aid. While the use of the phonological loop of working memory is involved in the inner search mechanism I previously described, we believe that a mechanism based purely on strategic use of working memory could explain the activation of the mouth motor system in recognition and recall tasks, but not in simple processing and categorization tasks. I am, therefore, more inclined to think that a monitoring process precedes the search for meaning occurring through inner speech (and working memory). However, further research is needed to disentangle which mechanism (or mechanisms) is primarily responsible for the mouth motor system activation.

### Differences across languages

So far, I have illustrated the role of language, considered as an inner and social tool, for abstract concepts. Importantly, however, we should consider not only language but languages, addressing the influence of cultural and linguistic diversity on cognition. Evidence will have to be found outside the WEIRD circuits (Henrich et al., 2010). Addressing this issue in depth is clearly out of scope here. Importantly, however, crosslinguistic evidence is particularly crucial for abstract concepts: the effects of linguistic variation are likely stronger for abstract than for concrete domains since the constraints provided by the environment are weaker (Borghi & Binkofski, 2014). Consistently, various studies have demonstrated big cross-linguistic differences with abstract concepts, such as that of time (e.g., Casasanto & Boroditski, 2008), while with concepts of objects, differences in naming less frequently correspond to differences in knowledge (e.g., Malt et al., 1999) (review: Borghi, 2019). In a recent study comparing Italian and Iranian participants, we found no evidence of a concreteness effect in Persian, likely because Persian is a poetic language, in which abstract metaphors are perceived as more concrete than in other languages (Ghandhari, Fini, Darold, Borghi, 2020). In sum: future research should consider more the impact of cultural and linguistic differences, and this need is particularly immediate for more abstract concepts see Mazzuca et al., 2019; 2020, on the concept of gender across languages.

### Kinds of abstract concepts

So far, I have spoken of abstract and concrete concepts, even if admitting that such a distinction can be a simplification. Literature has typically considered the broad distinction between these two categories and eventually investigated differences within concrete concepts, such as that between artifacts and natural objects (Keil, 1989) or between living and not living beings (Warrington & Shallice, 1984), and food (Rumiati & Foroni, 2016). However, recent research has demonstrated that different kinds of abstract concepts have partially different neural bases and elicit different behaviors. Evidence has shown that, compared to other abstract concepts, mental states engage more the mouth motor areas than other abstract concepts, such as numbers (Ghio et al., 2013; Dreyer & Pulvermuller, 2018); studies reported that numbers, at least low ones, are a special kind of abstract concepts, engaging the hand (finger counting experience) (Fischer & Shaki, 2018; Fischer & Brugger, 2011) primarily; further studies focused on the peculiarity of abstract social concepts (Mellem et al., 2015) and evaluative aesthetic concepts (Fingerhut & Prinz, 2018). Harpainter et al. (2018) found with a feature listing task that abstract concepts cluster into different groups. In a recent study, Desai et al. (2018) examined through neuroimaging meta-analyses the neural basis of four types of abstract concepts, numerical concepts, emotional concepts, morality judgements, and theory of mind: they found that they engage overlapping but also different brain areas. Based on the previously described ratings, we recently found that abstract concepts cluster into 4 different groups (Villani et al., 2019). They are: “philosophical-spiritual concepts” (e.g., value), which are more abstract, “physical, spatio-temporal and quantitative concepts” (e.g., reflex, addition, subtraction), for which sensorimotor information has higher weight, “self-sociality concepts” (e.g., politeness), and “emotive/inner states” (e.g., anger), grounded primarily in inner experience. In a further ongoing study (Villani et al., 2021) we found that different dimensions subtend different kinds of abstract concepts: for example, emotional concepts are more grounded in interoception than other abstract concepts. In sum, these studies demonstrate that the panorama of abstract concepts is richer and more heterogeneous than previously supposed, and future research should investigate more in-depth their differences.

### New methods

The new theories that highlight the importance of language and sociocultural practice for abstractness require rethinking the methods adopted to study them. Not only it is important to underline how the linguistic and social dimensions influence abstract concepts, but their social/interactive context in which they are used, and how they are used, should be taken into account. Current studies typically focus on single words, or simple sentences and adopt non-interactive paradigms. The necessity to develop new methods that capture concept and word “use in situated action” (Barsalou et al., 2018) but also in situated interaction, is particularly urgent for more abstract ones: the environmental constraints on their meaning are less powerful while the influence of the linguistic, pragmatic and social context is more pronounced. New methods are therefore necessary that take into account contextual variability and that capture conceptual use in the dynamics of real dialogues and conversations. The importance of studying them in dialogue is also justified by the fact that some underlying mechanism might change; for example, the degree of confidence resulting from the monitoring process at play during abstract words production and comprehension might differ. Novel behavioral and neuroscientific paradigms are emerging that should be merged with insights from pragmatics, semiotics, and linguistics. An example is paradigms taken from research in joint action (e.g., Knoblich et al., 2011; Fusaroli et al., 2012). In neuroscience, naturalistic fMRI methods adopting a social, or two-brain approach, allow investigating behavioral and neural processes in real-time-like social interactions (Nummenmaa et al., 2012). For example, Smirnov et al. (2019) studied neural speaker-listener synchronization during the narration of autobiographic emotional or neutral stories, that is using abstract emotional concepts.

### A grounded theory that ascribes a significant role to language and sociality

So far, I have tried to clarify why it is essential to adopt theories that a. are grounded and b. ascribe crucial importance to language and sociality. In this final section, I summarize some of the main findings on abstract concepts, most of which have been previously illustrated, and explain why they can be used to support such theories. The list is confined to behavioral findings and has no pretense of being exhaustive. As argued before, further evidence obtained adopting new, more ecological methods and not focusing on single words could contribute to a better understanding of conceptual use (Barsalou, 2020).

1. Concreteness effect, i.e., advantage of concrete over abstract concepts in processing, recognition and recall (Paivio, 1990). This is a robust finding, even if it was not present in some studies in which emotional valence was controlled (Kousta et al., 2011). Initially, it was explained based on imageability (Paivio, 1990) and contextual availability (Schwanenflugel et al., 1992); it has recently been accounted in terms of perceptual strength (Connell et al., 2012). It can, therefore, be explained by a grounded theory.
2. Role of interoception: ratings (Connell et al., 2018), and interference paradigms (Villani et al., under review) have highlighted that interoception is crucial for abstract concepts, particularly for emotional ones. This finding can be accounted for by an embodied theory that takes into account not only the role of sensorimotor features but also of inner grounding.
3. Introspection and word associations. Production tasks, such as the feature listing task, have shown that, compared to concrete concepts, abstract ones elicit with more word associations, more introspective/affective, and more social properties (Barca et al., 2017; Barsalou & Wiemer-Hastings, 2005; Harpaintner et al., 2018). Similarly, a recent study with the taboo task has revealed that, compared with concrete concepts, abstract concepts elicit more introspective properties and words referring to people, accompanied by less iconic and more metaphoric gestures (Zdrazilova et al., 2018). These findings can be accounted positing an important role for inner grounding and language and sociality in the representation of abstract concepts.
4. AoA, MoA etc. in rating tasks: recent rating tasks have shown that abstract concepts are associated to later Age of Acquisition than concrete concepts, linguistic instead of perceptual Modality of Acquisition, and a higher need for others to understand word meaning (social metacognition) (e.g., Villani et al., 2019). This finding can be explained by positing an important role of language and sociality for acquisition, representation, and use of abstract concepts.
5. Emotionality and valence: Recent evidence has shown that abstract words acquired early are emotionally valenced and that children acquire earlier emotional abstract concepts than other abstract concepts (Ponari et al., 2018; Lund et al., 2019). This finding can be explained by positing a critical role of emotional experience for acquisition, representation, and use of abstract concepts.
6. Mouth motor system activation: Evidence of such activation has been found in a variety of behavioral tasks in children and adults when comparing abstract with concrete concepts (acquisition of artificial categories, definition matching task, recognition task, interference paradigms, not found in lexical decision, studies on the use of a pacifier; for a review see Borghi et al. 2019). Furthermore, rating tasks (Ghio et al., 2013) and fMRI studies (Dreyer & Pulvermueller, 2018) have demonstrated a stronger activation of the mouth motor system with mental state abstract concepts. This finding can be explained by positing an important role of language for the acquisition, representation, and use of abstract concepts.
7. Differences across languages. There is evidence showing that abstract concepts differ more across languages than concrete concepts (review in Borghi, 2019). This can be explained by positing a more critical role of linguistic (and cultural) experiences for abstract than for concrete concepts.
8. Differences between kinds of abstract concepts (Crutch et al., 2013; Desai et al., 2018; Dreyer & Pulvermueller, 2018; Fischer & Shaki, 2018; Fingerhut & Prinz, 2018; Harpaintner et al., 2018; Mellem et al., 2016; Villani et al., 2019). Behavioral and fMRI evidence has shown that different kinds of abstract concepts differently recruit sensorimotor, affective, linguistic, social, theory of mind brain areas and experiences. Multiple representation views can account this finding, arguing that concepts are embodied but that also linguistic, affective, social experiences concur in abstract concepts representation.

This evidence, which is a subset of the existing one, points to the necessity of a theory of abstract concepts that is grounded but takes seriously into account the role of language. To do it, we need to go beyond theories that highlight only the role of the body and sensorimotor experience for cognition. Theories of distributional semantics, which emphasize the role of linguistic associations, might offer paramount insights to investigate how we represent abstract concepts. However, we think that this is not sufficient: language is a social and interactive experience, and it can change our social and physical environment. Pragmatics and semiotics can help us to capture this, providing us with methodological instruments suitable to study abstract words during natural interactions (see the section on new methods). Abstract concepts vary consistently across languages, more than concrete concepts. Anthropological and linguistic studies can help us to detect these variations. It could be objected that it is not economical to focus on many different aspects of language when investigating its impact on abstract concepts. But language is a complex phenomenon, and we think that it is high time to attempt to examine it in its multifaceted dimensions.

### Summary

To address the challenge of explaining abstract concepts from an embodied perspective, profound theoretical and methodological modifications should be introduced. The challenge implies a variety of changes that future research should face. The first and more crucial, in my opinion, consists of re-evaluating the role linguistic experience has in shaping our mind, recognizing the role language might have, especially as an inner tool and a social tool. The second is the necessity to study their variations across cultures and languages, investigating their use not only in WEIRD but also in other populations. Because they are less constrained by the correlational structure of the environment (Malt & Wolff, 2010), abstract concept are likely more influenced by the linguistic variation than concrete concepts. To address this challenge, anthropological studies that investigate how abstract concepts vary across languages are crucial. The third change consists in the recognition that abstract concepts come in different varieties and that it is necessary to investigate their fine-grained differences both in terms of content and of underlying mechanisms. The fourth, and final change, consists of introducing profound methodological modifications in the way to study them. Because abstract concepts are more variable across linguistic contexts, they should be investigated in the context of situated interaction (Barsalou et al., 2018), and because the social dimension is so crucial for them, they should be studied in the context of social interactions (Falandays & Spivey, 2019). In this respect, recent studies in pragmatics and psychological and neuroscientific literature starting to study language in situated, real-time interaction, offer very promising avenues.

## Conclusion

In this paper, I have tried to clarify why, in my view, understanding the capability for abstractness represents a crucial challenge. It is critical in general because explaining how we build and easily use difficult, abstract words, implies understanding one of the most essential human abilities. It is also particularly crucial for embodied and grounded cognition, because it may contribute to demonstrate that these theories hold not only for concrete, manipulable object and action verbs, but have a more extended reach. In this paper, I argue that, more than in the case of other words, dealing with abstract ones allows us to understand the importance linguistic experience has for cognition. As clarified in the introduction, language is a pointer and a shortcut to meaning, but it has other multifold and precious functions: it can work as a tool that modifies our perception of the physical environment, as an inner tool that improves our thinking capabilities, and as a social tool, that contributes to change our social setting. In this article, I have used abstract concepts as an example of a case in which language is particularly crucial, as it strongly constrains acquisition, representation, and use of concepts. I think one of the most important future challenges for embodied cognition is to highlight the pivotal role of language, capturing its flexibility, avoiding to contrast linguistic and embodied experience, and ascribing to it the role for our mind that it deserves. To address this challenge, an integrated approach is necessary. This approach should bridge behavioral, neuroscientific, and developmental evidence and extend embodied and grounded views incorporating insights from distributional views of meaning, pragmatics and semiotics. I would like to close with a quote from Virginia Wolf (The Death of moth and other essays): “Words… are the wildest, freest, most irresponsible, most unteachable of all things. Of course, you can catch them and sort them and place them in alphabetical order in dictionaries. But words do not live in dictionaries; they live in the mind…. They hang together, in sentences, in paragraphs, sometimes for whole pages at a time. They hate being useful; they hate making money; they hate being lectured about in public. In short, they hate anything that stamps them with one meaning or confines them to one attitude, for it is their nature to change.”
